# Worldwide Well-Being: Simulated Twins Reveal Genetic and (Hidden) Environmental Influences

**DOI:** 10.1177/17456916231178716

**Published:** 2023-06-29

**Authors:** Espen Røysamb, Terrie E. Moffitt, Avshalom Caspi, Eivind Ystrøm, Ragnhild Bang Nes

**Affiliations:** 1 Promenta Research Center, Department of Psychology, University of Oslo; 2 Norwegian Institute of Public Health, Oslo, Norway; 3 Department of Psychology and Neuroscience, Duke University; 4 Social, Genetic, and Developmental Psychiatry Centre, Institute of Psychiatry, Psychology, and Neuroscience, King’s College London; 5 Department of Philosophy, Classics, and History of Arts and Ideas, University of Oslo, Norway

**Keywords:** well-being, genetics, twins, environment, simulation

## Abstract

What are the major sources of worldwide variability in subjective well-being (SWB)? Twin and family studies of SWB have found substantial heritability and strong effects from unique environments but virtually no effects from shared environments. However, extant findings are not necessarily valid at the global level. Prior studies have examined within-countries variability but did not take into account mean differences across nations. In this article, we aim to estimate the effects of genetic factors, individual environmental exposures, and shared environments for the global population. We combine a set of knowns from national well-being studies (means and standard deviations) and behavioral-genetic studies (heritability) to model a scenario of twin studies across 157 countries. For each country, we simulate data for a set of twin pairs and pool the data into a global sample. We find a worldwide heritability of 31% to 32% for SWB. Individual environmental factors explain 46% to 52% of the variance (including measurement error), and shared environments account for 16% to 23% of the global variance in SWB. Worldwide, well-being is somewhat less heritable than within nations. In contrast to previous within-countries studies, we find a notable effect of shared environments. This effect is not limited to within families but operates at a national level.

What makes the worldwide population of 7.8 billion humans differ in subjective well-being (SWB)? What roles do genes and unique and shared environments play? Well-being is a fundamental human value (Diener et al., 2018a; Huppert, 2014) and a UN Sustainable Developmental Goal (Patel et al., 2018) and predicts important life outcomes (Diener et al., 2018a; Lyubomirsky et al., 2005; Sadler et al., 2011). Conceptually, SWB reflects evaluations of life as good or not so good and is typically operationalized by terms such as “life satisfaction” and “positive affect” (Diener et al., 2018a; Helliwell & Aknin, 2018). A deeper understanding of the genetic and environmental sources of worldwide variability in SWB, including a focus on country-level factors, may have considerable ramifications for well-being theories and the design and implementation of social policies and preventive and promotive measures. In a rapidly changing world, with war, pandemics, and climate change, there is an urgent need for more scientific knowledge on the nature and sources of human well-being.

Humans have migrated around the globe for thousands of years, and the current global population lives in a wide variety of contexts and life conditions. People may live as a nurse in Kyiv, a refugee in Raipur, a hunter in Xingu, or a barber in Seville. Each and every one has a particular genetic makeup and some unique life experiences and exposures and shares certain environmental factors with other people (Boomsma et al., 2002; Polderman et al., 2015). Two random people may be genetic peers in the sense of having the same genetic disposition to a certain phenotype, such as well-being. They may also be environmental peers in the sense of having similar environmental risk and protective factors.

There is solid evidence for genetic influences on well-being. Twin and family studies have typically reported heritability estimates in the .20 to .50 range (Bartels et al., 2022; Nes & Røysamb, 2016; Røysamb & Nes, 2019), and meta-analyses have reported weighted average heritabilities of .32 to .40 (Bartels, 2015; Nes & Røysamb, 2015), with heritability representing the proportion of phenotypic variance accounted for by genotypic variance. These meta-analyses have also reported substantial nonshared, or unique, environmental influences but no evidence for shared familial environmental factors. This finding implies that environmental factors shared by twins or siblings (e.g., parenting practices, family socioeconomic differences, residence area, and local culture) have limited effects on well-being within a nation’s population.

Generally, behavioral-genetic studies have found little evidence of shared environmental factors in psychological traits. The notion that most environmental effects are unique rather than shared in families is listed among the top 10 replicated results in behavioral genetics: “The basic finding that most environmental effects are not shared by family members remains one of the most far-reaching findings from behavioral genetics” (Plomin et al., 2016, p. 13). Furthermore, Polderman et al. (2015) published a meta-analysis of heritability across 50 years of twin studies, including 2,748 studies covering more than 14 million partly independent twin pairs and 17,804 traits. For a majority of traits (mental and physical), the findings are consistent with a model in which twin resemblance is solely due to genetic factors, implying no effects of shared environments on making people similar to each other (Polderman et al., 2015). The finding of limited or negligible effects of shared environments is so pervasive that it has been coined the “second law of behavioral genetics” (Turkheimer, 2000) and is widely accepted across developmental science and psychology.

Another line of well-being research has focused on national differences in well-being and has reported substantial variability in mean levels across nations (Diener et al., 2018a; Helliwell, Layard, et al., 2020). The *World Happiness Report* (Helliwell et al., 2022; Helliwell, Layard, et al., 2020) provides annual rankings of life evaluations in countries worldwide. Typically, the Northern European countries score in the top tier, while countries burdened with war, poverty, and conflict report the lowest scores. On a scale from 0 to 10, national mean scores vary from around 2.4 in Afghanistan to 7.8 in Finland (Helliwell et al., 2022). How can findings of substantial heritabilities and no shared environmental effects be reconciled with these findings of large mean differences between countries? If countries have characteristics (e.g., economy, health policies, governance, crime rates, safety, armed conflict, cultural practices) that contribute to national mean levels of well-being, these should partly be shared by the inhabitants within each country.

One basic limitation of the reported heritability estimates for well-being is that they are based on within-countries variability. Typically, twin and family samples have provided opportunities for estimating genetic and environmental contributions to variance within countries but have not taken between-countries variability into account. Thus, extant evidence for genetic and environmental effects on well-being does not include the potential effects of environmental factors shared at the national level. Further knowledge on the sources of worldwide well-being, including country-level shared environmental factors, may have implications for understanding the nature of well-being and for efforts to promote well-being.

The shared environment is defined as all factors that contribute to similarity between twins and family members beyond the effects of genetics (Boomsma et al., 2002; Plomin et al., 2016). That is, the shared environment is identified by its consequences in terms of generating resemblance within families. We point out that the degree of similarity depends on the heterogeneity of the population in focus and that shared environments comprise both effects that are shared uniquely in families and effects that are shared by larger groups—including families. If there are putative factors shared by people in a certain area, the effect should show up as a shared environmental effect in behavioral-genetic studies (Tamimy et al., 2021). However, such effects have been detected to only a very limited degree so far. We argue that the apparent absence of shared environmental effects is due to homogeneity in the populations studied, possibly combined with limited statistical power. By using well-established twin methods, we aim to show that between-countries variability can be captured as a shared environmental effect that is not discovered in within-countries analyses.

The notion that between-countries mean differences represent a shared environmental effect may also be extended into regional differences within countries. Although twin studies generally have not identified such effects, other evidence points to region-wise mean differences (Helliwell et al., 2019; Helliwell, Shiplett, & Bonikowska, 2020; Lawless & Lucas, 2011). Currently, there is limited knowledge about the role of regional differences for global variability in well-being.

Twin and family studies are unique in their ability to capture total genetic and environmental effects for a given phenotype. Recently, molecular-genetic studies have shown exciting progress, also in the field of well-being (Baselmans et al., 2019; Rietveld et al., 2013), and genome-wide association studies have started to identify specific variants involved, currently explaining 1% to 2% of the variance (Okbay et al., 2016; Turley et al., 2018). Despite the progress in molecular genetics, the behavioral-genetic approaches, including twin, family, and adoption studies, hold several advantages. The beauty of this design lies in the power to detect the total genetic and environmental sources of variance without observing these factors directly. Based on a set of known relations (e.g., 100% and 50% shared genes for monozygotic [MZ] and dizygotic [DZ] twins, respectively) in combination with observed similarities for given phenotypes, the twin design enables identification of the entire genetic effect and also distinguishes between shared and nonshared environmental effects.

We aim to estimate the major sources of well-being worldwide to provide answers to these fundamental questions: What is the heritability of subjective well-being globally? To what extent do country-level factors represent shared environments that are overlooked in within-countries heritability studies? What are the contributions of shared and nonshared environments in generating the total variability of well-being for the world population?

Because there are no empirical data available to estimate sources of worldwide variability directly, we developed a strategy of combining existing estimates of within-countries heritability (Bartels, 2015; Nes & Røysamb, 2015) and national levels of means and standard deviations (Helliwell, Layard, et al., 2020) to simulate scenarios with worldwide twin samples and provide estimates of the main factors underpinning well-being. More specifically, we simulate twin studies in 157 countries and present estimates of the main sources of variability in well-being globally.

## Method

### Extant data: the knowns

Our strategy involves using sets of known parameters to estimate a set of unknown values. The known parameters include empirical evidence from the following two fields of research.

First, numerous twin and family studies have reported heritability estimates for well-being. One meta-analysis (Nes & Røysamb, 2015), based on more than 30,000 respondents in 13 separate studies, reported a weighted mean heritability of .40 for well-being. Another meta-analysis (Bartels, 2015), based on more than 55,000 respondents across 10 independent studies, reported a weighted mean heritability of .36 for general well-being.

Given the two highly convergent meta-analyses, we use a heritability of .38 (midpoint of .36 and .40) as our starting point. Because this is not a fixed statistic but, rather, a mean heritability with variability, we set out to model scenarios in which countries vary around this mean. Moreover, in addition to these estimates for subjective well-being in general, Bartels (2015) provided an estimate of .32 for the specific well-being component of life satisfaction. Hence, we also include a scenario based on this parameter estimate. Note also that the findings from the meta-analyses have been supported by more recent studies (Bartels et al., 2022; Jamshidi et al., 2020; Røysamb et al., 2018).

Second, the *World Happiness Report* (Helliwell, Layard, et al., 2020) provides country-wise annual summary statistics (means and standard deviations) for life evaluation, downloadable from its website. Data are based on population-based interviews, conducted by the Gallup World Poll, with an average sample of about 1,000 in each country. The data are collected with the Cantril Ladder (Cantril, 1965), in which respondents are asked to evaluate their lives on a scale from 0 to 10, from *worst possible life* (0) to *best possible life* (10). We used the reported means and standard deviations for each country as a best estimate of current levels and variability for the 5-year period of 2015 to 2019. A total of 157 countries had valid data for this period (i.e., for 1 or more years), representing 81% of all 195 countries. Note that the *World Happiness Report* publishes 3-year averages but also provides information about yearly scores. Finally, population sizes were downloaded from worldofmeters.info, with data from the United Nations Population Division estimates.

### Concepts and measures

In this study, we use “subjective well-being,” or more briefly, “well-being,” as a general term. Typically, SWB refers to an evaluation of life with cognitive and emotional components (Diener et al., 2003, 2018a). The twin studies included in the meta-analyses have used several measurements for well-being, including the Satisfaction With Life Scale, the Subjective Happiness Scale, the Quality of Life Scale, and the Life Satisfaction Index (Bartels, 2015; Nes & Røysamb, 2015). The *World Happiness Report* uses the Cantril Ladder (Helliwell et al., 2022). Although the scales used across studies are not identical, they all capture well-being. Some researchers have used the Cantril Ladder as a direct measure of life satisfaction (Ball & Robbins, 1986; Bjarnason et al., 2012), whereas others have noted an empirical difference between the Cantril Ladder and life satisfaction (when asked of the same respondents) but have found a high degree of consistency (Helliwell, Shiplett, & Bonikowska, 2020). While acknowledging differences and nuances between the scales, we believe it is fair to consider the included scales as valid measures of SWB.

Data suggest that humans across the globe tend to assess how much they like their life. This evaluation is linked to gratification of universal humans needs and based on comparisons framed by cultural standards of the good life (Veenhoven, 2010). Substantial attention has been paid to translational challenges, and studies such as the Gallup World Poll (*World Happiness Report*), the World Values Survey, and other national studies have provided evidence for the validity of cross-cultural comparisons (Diener et al., 2010; Helliwell et al., 2022; Inglehart & Klingemann, 2000; Krys et al., 2022; Vitterso et al., 2002). In total, the scales used are considered reasonably reliable and valid for comparisons both within and between countries.

### Twin correlations: estimates of heritability and environmental effects

Twin studies have the potential to estimate underlying causal factors by features of the design. Neither the genetic nor the environmental factors need to be observed; their effects are deduced from the cross-twin correlations for MZ and DZ pairs (Boomsma et al., 2002; Polderman et al., 2015). Three major factors are typically estimated: additive genetic effects (A), shared or common environment (C), and nonshared environment (E). The magnitude of effects is given by the following (Falconer’s) equations (Polderman et al., 2015):

$$A=a^{2}=h^{2}=2\left(r_{\mathrm{MZ}}\;-\;r_{\mathrm{DZ}}\right)$$

$$C=c^{2}=2r_{\mathrm{DZ}}-r_{\mathrm{MZ}}$$

$$E=e^{2}=1-r_{\mathrm{MZ}}.$$

Here, *r*_MZ_ is the twin–cotwin correlation for MZ pairs, and *r*_DSZ_ is the corresponding correlation for DZ pairs. These formulas provide precise values for the variance components, *A*, *C*, and *E*. Specifically, the heritability (*h*^2^) is given by twice the difference in the MZ versus DZ correlation. The shared environmental variance is given by twice the DZ correlation minus the MZ correlation. Thus, a C factor is indicated only when the DZ correlation exceeds half the MZ correlation. When the DZ correlation is less than half the MZ correlation, nonadditive genetic effects are indicated. Such effects include within-alleles effects (dominance) or between-alleles effects (epistasis; Polderman et al., 2015). Because the meta-analyses providing the input parameters for our simulations found little evidence for nonadditive genetic effects, we focus on the A, C, and E components.

In empirical twin studies, biometric models and structural equation modeling (OpenMx, Mplus) are typically used to obtain fit measures, confidence intervals (CIs), and comparison of alternative models and to test more complex multivariate models. For our purposes, the Falconer’s formulas (above) are sufficient and provide for transparent communication of results.

### Analytic strategy: simulations

We simulated twin data for each of the countries with known means and standard deviations in the Gallup World Poll (Helliwell, Layard, et al., 2020). For MZ twin pairs, we created two random variables correlated at *r* = .38 and with means and standard deviations as reported for the specific country. Correspondingly, similar variables were created for DZ twin pairs, but with a twin–cotwin correlation of .19. The correlations of .38 and .19 are based on the equations above; that is, these would be the observed correlations that result in the extant heritability estimates (i.e., A = .38, C = 0, E = .62). Most analyses were conducted for averages (of means and standard deviations) over 5 years (2015–2019), but we also provide estimates for specific years.

The simulated data for all countries were pooled to provide a data set mimicking the scenario of having worldwide twin data. Next, we calculated twin–cotwin correlations for MZ and DZ pairs in this worldwide data set. Finally, this simulation was repeated 100 times (as a Monte Carlo simulation) to reduce random effect and obtain precise parameter estimates and standard errors.

To examine the robustness of findings, we simulated five scenarios involving different assumptions and input parameters. First, in our basic scenario, we simulated samples of 1,000 MZ and 1,000 DZ twin pairs in each country (i.e., total sample size of 314,000) and used A = .38 and E = .62 as input parameters. Second, to account for the differences in country sizes, we adjusted sample sizes in each country to reflect their proportion of the global population. Third, we included a C effect to reflect the presence of regional differences within countries in addition to the between-countries differences. We also provide results for each year. Fourth, we tested a scenario with within-countries heritability = .32 (rather than .38), based on the meta-analytic findings of the specific well-being component of life satisfaction. Finally, we adjusted for random measurement error, which typically is contained in the E-parameter, and provide corresponding results. All R code for the simulations can be found in the Supplemental Material available online, along with an elaboration of technical aspects of setting up the twin data.

## Results

### Data overview

Table 1 shows an overview of the data input and output of the simulation analysis. The left side represents known parameters based on the existing literature. The right side shows a simplified version of the simulated data for both twins in a pair.

**Table 1.** Overview of Input Parameters and Simulated Data for Well-Being

Input parameters				Simulated data			
Country	Country *M*	Country *SD*	Correlation Twin1–Twin2	Twin pair	Zygosity	Well-being Twin 1	Well-being Twin 2
1	3.2	1.7	*r* ≈ .38 (MZ)	1	MZ	1.7	2.8
				2	MZ	2.9	4.0
				. . .	MZ	. . .	. . .
				1,000	MZ	3.1	2.9
			*r* ≈ .19 (DZ)	1	DZ	1.4	3.6
				2	DZ	5.9	5.0
				. . .	DZ	. . .	. . .
				1,000	DZ	4.1	5.9
2	4.8	2.7	*r* ≈ .38 (MZ)	1	MZ	5.1	6.2
				2	MZ	4.9	4.0
				. . .	MZ	. . .	. . .
				1,000	MZ	5.3	6.9
			*r* ≈ .19 (DZ)	1	DZ	4.7	3.8
				2	DZ	5.9	8.0
				. . .	DZ	. . .	. . .
				1,000	DZ	6.1	4.9
3	5.1	2.0	*r* ≈ .38 (MZ)	1	MZ	3.7	4.8
				2	MZ	5.9	7.0
				. . .	MZ	. . .	. . .
				1,000	MZ	5.1	3.9
			*r* ≈ .19 (DZ)	1	DZ	3.7	4.8
				2	DZ	2.9	6.0
				. . .	DZ	. . .	. . .
				1,000	DZ	7.1	4.9

Note: Table presents simplified overview of data matrix. Total *N* (countries) = 157; *N* (twin pairs per country) = 2,000; Total *N* = 314,000. MZ = monozygotic; DZ = dizygotic.

**Fig. 1.**
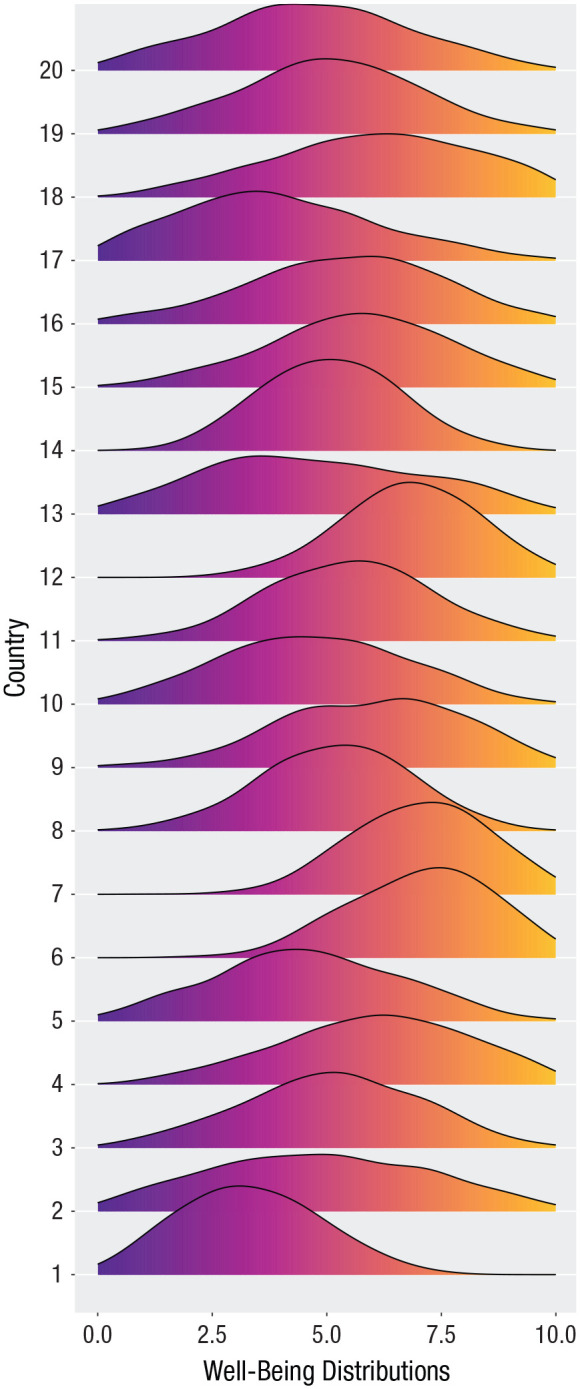
Distributions of well-being for 20 countries.

Figure 1 shows the simulated distribution of well-being for a sample of countries, that is, the 20 first, alphabetically, from Afghanistan to Burkina Faso (for full list, see the Supplemental Material). Simulations were set up so that the means and standard deviations were identical to the empirical values for each country. As is shown, there are cross-country variability in both means and variances.

### Twin correlations

**Fig. 2.**
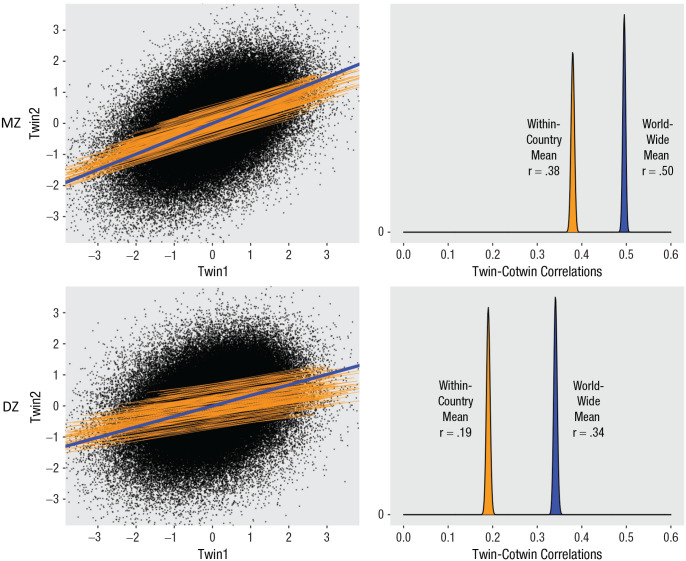
Association of well-being for Twin1 and Twin2. Scatterplot (left) with slopes for each country (orange) and worldwide (blue), for monozygotic (MZ) twins (upper) and dizygotic (DZ) twins (lower). Standardized scores. Corresponding distribution plots (right) for within-countries (orange) and worldwide (blue) correlations, for MZ (upper) and DZ (lower), across 100 simulations. Shown values (e.g., .38) are mean r’s across simulations.

Figure 2 (left) shows associations between Twin1 and Twin2 for MZ (upper) and DZ (lower) twins. The orange lines represent slopes for each country, which are set to *r* ≈ .38 (MZ) and *r* ≈ .19 (DZ). The left-side figures are from one simulation, and all repetitions yielded similar images. Because there is randomness inherent in the simulated data, the MZ *r*s varied between .28 and .48 across countries (i.e., average minimum and maximum in one simulation). Correspondingly, *r*s for DZ twins varied between .06 and .31. This variability mimics the variation in heritabilities across samples and countries and is manifested in the lines not being perfectly parallel. The blue lines (left) show associations for the pooled worldwide sample. As is shown, the slopes are steeper than the corresponding within-countries lines for both zygosity groups.

Figure 2 (right) shows the distribution of within-countries and worldwide mean correlations for MZ (upper) and DZ (lower), corresponding to the lines in the left side of the figure (and with corresponding color code). Whereas the left side is based on one simulation, the right side shows the distributions across all 100 simulations. The distribution for within-countries correlations is based on the average correlation (across all countries) for each simulation.

For MZ twins, the estimated worldwide twin–co-twin correlation *r*_w_ was .50, and the corresponding correlation for DZ twins was .34. Thus, both the MZ and DZ correlations increased compared with the initial values of .38 and .19, respectively. Note also that the worldwide DZ correlation exceeds half the MZ correlation, implying the presence of a C factor, unlike in the within-countries data.

### Estimated variance components

**Fig. 3.**
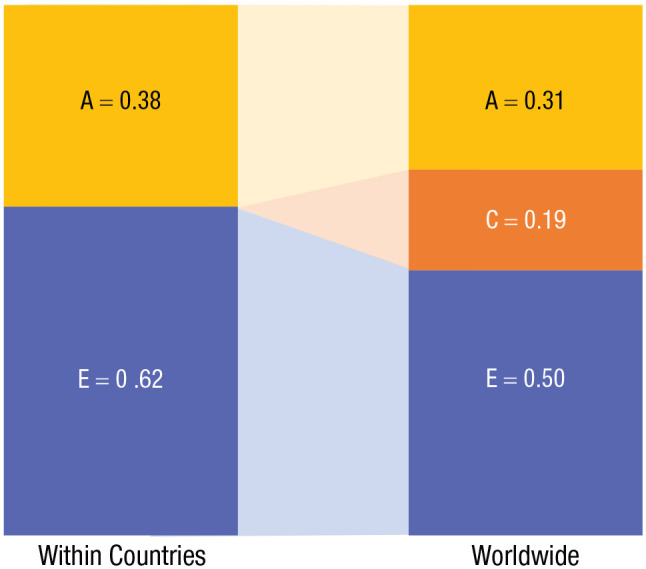
Environmental and genetic variance components. Estimated effects (variance components) due to A (additive genetic factors), C (shared environment), and E (nonshared environment), within countries (left) and globally (right) in the period 2015 to 2019.

On the basis of the estimated correlations, we calculated the implied A, C, and E components. Figure 3 shows the variance in well-being explained by A, C, and E when analyzed within countries (left) and worldwide (right). We estimate that international differences in shared environments explain 19% of the global variance, with the shares of heritability and unique environments being correspondingly reduced from their typical within-countries shares. Full estimates were A = .31 (95% CI = [.30, .32]), C = .19 (95% CI = [.18, .19]), and E = .50 (95% CI = [.50, .51]).

### Sensitivity analyses: modeling alternative scenarios

To examine the sensitivity and robustness of findings, we conducted analyses reflecting different scenarios.

#### Scenario 2

The initial analyses (above) are based on a situation in which we sampled 1,000 twin pairs (for each zygosity) in every country and then merged the data sets into a worldwide sample. However, countries vary in population size and represent different proportions of the total world population. Thus, we simulated a scenario in which sample sizes varied across countries to reflect each country’s proportion of the global population. To retain comparability with the first set of analyses, we used the same total sample size.

The results were highly similar to those obtained in the initial unweighted analysis: A = .32 (95% CI = [.31, .33]), C = .16 (95% CI = [.15, .17]), and E = .52 (95% CI = [.52, .52]). Thus, when taking into account the different population sizes, we observed a slightly higher genetic effect and slightly lower shared environmental effect but generally converging findings.

#### Scenario 3

The two previous scenarios are both based on the absence of a C factor in prior (within-countries) twin studies. However, given limited power to detect small C effects in typical sample sizes, such effects could theoretically still be present. In addition, there are other national studies that have reported within-countries regional differences, which would imply a certain C factor (Helliwell et al., 2019). Therefore, we modeled a scenario specifying a within-countries C = .05. The exact magnitude of this C is difficult to determine based on existing evidence, but this level would correspond to the notion of regional differences being smaller than country differences and not detectable in twin studies with limited sample sizes. Thus, the input parameters (within-countries) were A = .38, C = .05, and E = .57.

The worldwide simulation yielded the following results: A = .31 (95% CI = [.30, .32]), C = .23 (95% CI = [.22, .24]), and E = .46 (95% CI = [.46, .47]). Again, we see the same level of genetic effects as in the previous scenarios, an added effect from shared environments, and slightly reduced effect from unique environments.

**Fig. 4.**
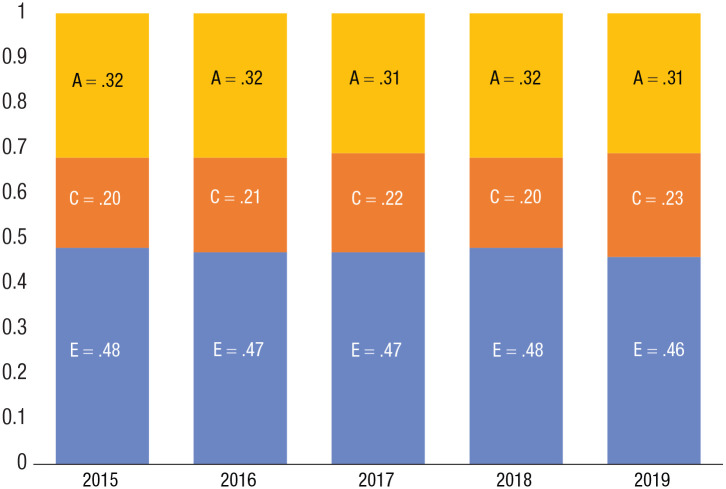
Environmental and genetic variance components. Worldwide parameter estimates for additive genetic factors (A), shared environments (C), and unique environments (E), for each year (2015–2019), taking into account unequal population sizes across countries and regional differences within countries.

Adding to the robustness, we also repeated the simulations of this scenario with separate analyses for each year in the observation period. Figure 4 shows the results, with modest variations in parameter estimates: A = .31 to .32, C = .20 to .23, and E = .46 to .48.

#### Scenario 4

In Scenarios 1 through 3, we used A = .38 and E = .62 as within-countries input for the simulations based on the meta-analyses of subjective well-being. Because one meta-analysis (Bartels, 2015) also included separate estimates for the specific component of life satisfaction (i.e., A = .32), we specified a simulation based on these parameters (with the population size adjustment, as in Scenario 2). The worldwide results were A = .27 (95% CI = [.26, .28]), C =.16 (95% CI = [.15, .17]), and E = .57 (95% CI = [.57, .57]).

#### Scenario 5

Finally, we recalculated the A, C, and E components while adjusting for measurement error, again taking Scenario 2 as a starting point. Random measurement error is included in the E component and needs to be subtracted to provide adjusted estimates. Given a reliability of around .80 for the well-being measures (Diener et al., 2018b; Levin & Currie, 2014; Pavot & Diener, 2008), the worldwide estimates, averaged across the time span, were: A = .40 (95% CI = [.38, .41]), C = .20 (95% CI = [.19, .21]), and E = .40 (95% CI = [.39, .40]).

To summarize, for all the alternative scenarios, we observed two key findings: Compared with extant within-countries findings, a worldwide perspective on well-being leads to (a) identification of a shared environmental factor explaining around 20% of the variance and (b) a corresponding reduction in heritability and effects from the nonshared environment.

## Discussion

We set out to examine the major sources of worldwide variability in SWB. To what extent are differences in human well-being due to genetic differences, shared environmental factors, or unique environmental factors? Previous genetically informed studies on well-being have shown substantial genetic effects, and meta-analyses have reported weighted average heritabilities of .36 to .40 for subjective well-being in general (Bartels, 2015; Nes & Røysamb, 2015). Environmental effects are consistently found to be important, more so than genetic effects, but appear to be unique to each individual and not shared by families and groups of people. The somewhat counterintuitive finding of no shared environmental effects for well-being accords with general findings for most mental and physical traits in adults (Polderman et al., 2015). We argue that despite their high value, twin studies have primarily examined within-countries variability. To address important questions about humankind in general, researchers need to expand the temporal-spatial dimension and take into account variability both within and between countries.

The current analyses revealed a worldwide effect of the shared environment accounting for 16% to 23% of the variance in well-being, corresponding to betas of 0.40 to 0.48 as regression coefficients. This finding contrasts prior findings of virtually no shared environmental effects (Bartels, 2015; Nes & Røysamb, 2015). We argue that previous studies are valid and important but mainly concerned with the variance within a country—or sometimes only regions of nations. Thus, there is substantial homogeneity in environmental exposures in previous studies. When expanding the scope, considering the current world population, and capturing the heterogeneity of life conditions, a notable effect of shared environments is revealed.

Shared environments are defined as everything that contributes to similarities between twins or other family members (Polderman et al., 2015). These factors may operate at several levels and may be unique to families or to larger groups. To be identified in twin and family studies, it is required only that they cause similarity relative to the entire population in focus. Our findings suggest that shared environments, as manifested in similarities within nations, represent a substantial source of variability in well-being in a global perspective. Our analyses cannot disentangle the specific national factors that contribute to the national aggregation of well-being. Yet factors such as trust, health-care systems, national economy, distribution of wealth, cultural value systems, governance, corruption, war, and conflict are potential candidates (Diener et al., 2010; Helliwell et al., 2022).

Statistically, our findings may serve as a proof of concept that between-countries mean differences will come out as a shared environment factor in twin analyses when data sets are pooled into a worldwide sample. We believe this may be obvious to some but unknown to many. Across the different models tested, the shared environmental effect was around 20%. The robustness of this effect is not a chance finding but, rather, due to the proportion of between-nations to within-nations variance, and similar figures may be obtained by direct calculations. A main advantage of performing a simulation study of twins is to put the findings in a context of previous genetically informative studies and to translate the between-countries differences into a component of shared environmental variance. Future research should seek to collect twin data from more countries and cultures to provide empirical evidence for the role of country-level factors.

The need for joint consideration of group means and variances in behavior-genetic studies was recently pointed out (Burt & Johnson, 2023). However, we note that the identification of a shared environmental factor reflecting group mean differences does not inform one of its origin. Some shared factors might in fact be due to genetic ancestry, while others, as we argue for well-being, will be environmental in nature. For example, given that there are country differences in mean height, a pooled twin analysis would yield a shared environmental factor. Nevertheless, this factor might in reality be partly driven by genetic differences. In the case of well-being, we know of no studies showing a substantial effect of genetic factors on mean country levels. On the contrary, studies of migration have shown immigrants to end up with well-being levels around the level of their new country rather than their country of origin (Helliwell, Shiplett, & Bonikowska, 2020), thus testifying to the environmental origin of country-level well-being. Overall, we believe it is fair to conceive of between-countries effects as basically environmental.

The twin meta-analyses used as input (i.e., within-countries studies) did not reveal any shared environment factor. However, other evidence (Helliwell, Shiplett, & Bonikowska, 2020; Lawless & Lucas, 2011) points to regional differences in well-being within countries. Such differences are expected to show up as a C factor in twin studies. We argue that a small C factor could be present without being identified in some twin studies because large samples generally are required for sufficient power. In our third scenario, we included a small within-countries C factor representing regional differences. The exact magnitude of this factor is uncertain, but we believe inclusion of this perspective is important, and the simulation serves as an example of results given plausible input parameters.

We found that worldwide well-being is somewhat less influenced by genetic factors than what is found within nations. This is not surprising given that heritability is a relative variance statistic, referring to the genetically caused variance as a proportion of total variance (Boomsma et al., 2002). As the total variance increases, by including between-countries variance, the genetically explained variance will represent a smaller fraction. Nevertheless, when adjusting for random measurement error (Scenario 5), we found a worldwide heritability of .40. Because error adjustment has the potential to provide true estimates, we believe this figure plausibly reflects reality. Yet in most of the modeled scenarios, we did not control for measurement error and believe this approach provides for direct comparisons with previous within-countries studies, also conducted without such control.

The notion of heritability as a relative statistic should be understood in contrast to the concept of absolute genetic variance. If the environmental variance increases and the absolute genetic variance remains the same, the heritability decreases. In a recent study, van de Weijer et al. (2022) showed increased environmental variance in quality of life during the COVID-19 pandemic. Whereas the absolute genetic variance remained similar, the heritability was reduced. Our analyses focused on the mean heritability from the meta-analyses as providing a best estimate for (within-countries) heritability worldwide rather than assuming a constant absolute genetic variance. More research is needed to address the issue of relative versus absolute genetic variance similarities across countries.

Across the models, the nonshared environmental factor was a large contributor to well-being worldwide. That is, despite substantial effects of national-level shared environments and individual genetic factors, a main source of well-being is still the unique experiences of each individual. These experiences are not shared with fellow citizens of one’s country or with one’s (hypothetical) MZ co-twin and contribute strongly to human well-being. Thus, twin studies represent a design that provides compelling evidence for the causal effects of environments and unique life stories.

What are the implications of our approach beyond the field of well-being? The logic of country-level factors operating as a shared environment is valid across traits and disorders. Yet we would expect to find a similar presence of shared environmental factors only for conditions showing country-level differences in means or prevalences. For example, if suicide rates differ across countries but schizophrenia prevalences do not, only the former would come out with a shared environmental component in a worldwide perspective.

The notion of geographic, country-specific clustering of shared environmental effects may also be translated to a notion of similar effects across time. For example, for adult intelligence, studies have reported substantial heritability and limited effects of shared environments (Boomsma et al., 2002; Polderman et al., 2015). However, there has been a substantial increase in IQ scores across generations, often denoted as the “Flynn effect” (Flynn, 2018). This secular change in IQ would show up as a shared environmental effect in twin studies if a sufficient time span was included. Thus, expanding the spatiotemporal dimension into worldwide or time-wide perspectives would enable a more nuanced analysis of factors that contribute to human variability, ranging from genes to societal time-variant factors.

Our simulations and the twin meta-analyses they build on do not examine gene-environment interplay. Such interplay, including both correlations and interactions, is generally important across phenotypes and is increasingly being subject to study in well-powered samples. For example, a recent molecular-genetic study of socio-economy found evidence for gene-environment correlations involving polygenic scores and geographic regions (Abdellaoui et al., 2022). Another recent study revealed interactions between attention-deficit/hyperactivity disorder-related genotypes and school quality in predicting academic achievement (Cheesman et al., 2022). Thus, gene-environment interplay involving regions or schools is clearly evident, and we expect to see more such studies coming.

For well-being, our findings of a worldwide C factor raise a set of new questions about how genetic dispositions interact with culture, national economy, governance, and other country-level factors (G × C interaction). For example, cultures may differ in how they value different human characteristics (e.g., extraversion, body mass, school performance). Consequently, to the extent that these features are genetically influenced, the specific genetic factors contributing to well-being may vary across these cultures. Moreover, countries may differ in the extent to which they provide opportunities for genetic potentials and resources to be expressed and developed. For example, providing equal opportunities for education may allow genetic potentials for learning to flourish and hence potentially contribute to well-being for individuals and societies.

There are some limitations inherent to the current study. First, our simulations are based on recent empirical evidence from two main sources: meta-analyses of twin studies (Bartels, 2015; Nes & Røysamb, 2015) and national mean levels and standard deviations of well-being (Helliwell, Layard, et al., 2020). The twin studies are mainly conducted in Western, industrialized countries and are not necessarily representative for all countries. Correspondingly, the national mean-level data represent most but not all countries and may not include fully representative samples. Yet these data sources represent the current state of knowledge and thus provide the best available input. Second, our main simulations involve assumptions of genetic and environmental effects operating in a proportional manner, and these assumptions may not be fully correct. However, by including analyses reflecting different scenarios, we show that the main findings remain across several assumptions. Third, the input parameters are based on studies of adults. Hence, our findings do not necessarily pertain to the well-being of children. Fourth, in our simulations, all twin pairs are assumed to live within the same country. In real life, there would be cases of twin pairs split in different countries, but we do not believe these relatively rare cases would substantially influence results. Finally, our analyses do not address sex differences, assortative mating, or associations with other phenotypes. These are all important topics but beyond the scope of this article.

In summary, previous studies have shown substantial heritability and virtually no effect of shared environmental factors on human well-being (Bartels, 2015; Nes & Røysamb, 2015). We argue that these findings are highly valuable and robust but limited to within-countries variability and not necessarily representative of global variation in well-being. With a worldwide perspective, we find notable effects of shared environments, which typically are not found in within-countries studies. That is, taking available data on the means and standard deviations of national well-being estimates into account raises by about 20% the total individual-level variance to be explained. Taking these international differences to represent the effects of nationally shared environments thus correspondingly reduces the shares contributed by other factors. The shared environment contributes to well-being similarity between twins and other family members but is not unique to each family. Rather, a key part of the shared environment lies in country-specific features and may thus be dynamic and changeable. Methodologically, our study shows that between-countries mean differences show up as a shared environment factor in twin modeling, and we hope our perspective and findings contribute to building a bridge between the behavior-genetic field and that of national comparisons of well-being.

In a changing world, with pandemics, climate change, political polarizations, and wars, we believe findings on key sources of worldwide variability in well-being provide information relevant to health authorities, policymakers, and governments. As a UN Sustainable Development Goal (Patel et al., 2018), well-being warrants high scientific attention, and by including a global perspective, researchers may learn about both the nature and promotion of well-being.

## Supplemental Material

sj-docx-1-pps-10.1177_17456916231178716 – Supplemental material for Worldwide Well-Being: Simulated Twins Reveal Genetic and (Hidden) Environmental Influences Click here for additional data file. Supplemental material, sj-docx-1-pps-10.1177_17456916231178716 for Worldwide Well-Being: Simulated Twins Reveal Genetic and (Hidden) Environmental Influences by Espen Røysamb, Terrie E. Moffitt, Avshalom Caspi, Eivind Ystrøm and Ragnhild Bang Nes in Perspectives on Psychological Science

## Transparency

*Action Editor:* Leonel Garcia-Marques

*Editor:* Interim Editorial Panel
