# Supplementary material for: Worldwide Well-Being: Simulated Twins Reveal Genetic and (Hidden) Environmental Influences
Source: Perspect Psychol Sci. 2023 Jun 29;18(6):1562–74. doi: 10.1177/17456916231178716 (PMC10623597; doi:10.1177/17456916231178716)
Supplement: sj-docx-1-pps-10.1177_17456916231178716 – Supplemental material for Worldwide Well-Being: Simulated Twins Reveal Genetic and (Hidden) Environmental Influences [file sj-docx-1-pps-10.1177_17456916231178716.docx]

**Supplementary Material**

**Worldwide wellbeing: Simulated twins reveal genetic and (hidden) environmental influences**

Røysamb, E., Moffitt, T. E., Caspi, A., Ystrøm, E., & Nes, R. B.

**Content of Supplementary Material**

1) Technicalities of the simulation. Code and rationale

2) Additional scenario

3) Extra figure

4) Country means and standard deviations

5) Full R code used for simulations

**1) Technicalities of the simulation**

The core part of the basic simulation (scenario 1) was conducted with the following code:

a1 <- 0.38 # Heritability (within-country)

c1 <- 0.0 # Shared environment (within-country)

e1 <- (1-a1-c1) # Unique environment (within-country)

n <- 1000 # N of cases (twin pairs, each zygosity) per country

## MZ twins

for(i in 1:length_yr) { #lenght_yr is number of valid cases (countries) for a given year/period

x <- rnorm(n,0,1)

y <- rnorm(n,0,1)

z1 <- rnorm(n,0,1)

z2 <- rnorm(n,0,1)

mz1[,i] <- as.vector(mwb[i] + sdwb[i]*scale(sqrt(a1)*x + sqrt(c1)*y + sqrt(e1)*z1))

names(mz1)[i] <- paste("m1_",i, sep="")

mz2[,i] <- as.vector(mwb[i] + sdwb[i]*scale(sqrt(a1)*x + sqrt(c1)*y + sqrt(e1)*z2))

names(mz2)[i] <- paste("m2_",i, sep="")

mz_cor[i] <- cor(mz1[i],mz2[i])

}

*Comments and elaboration*

- a1=genetic variance; c1= common/shared environment; e1=non-shared environment. The square roots of a1, c1 and e1 represent the path coefficients from the underlying factors onto phenotypic wellbeing. a1 and c1 are set at certain levels (e.g. .38 and .00) based on the meta-analyses
- x, y and z are underlying factors representing A, C and E, and are drawn from random normal distributions.
- mwb is a vector of means for all countries, based on the World Happiness Report (WHR)
- sdwb is a vector of standard deviations for all countries, based on the (WHR)
- mz1 and mz2 are matrices (1000 x 157), where individual scores for each person are stored
- mz_cor stores correlations between twin1 and twin2 (n=1000) for each of 157 countries
- The code “mz1[,i] <- as.vector(mwb[i] + sdwb[i]*scale(sqrt(a1)*x + sqrt(c1)*y + sqrt(e1)*z1))” creates scores for a given person so that her score is based on random variables x, y and z with effects= square roots of a1, c1 and e1, times the sd of her country, around her country mean.
-
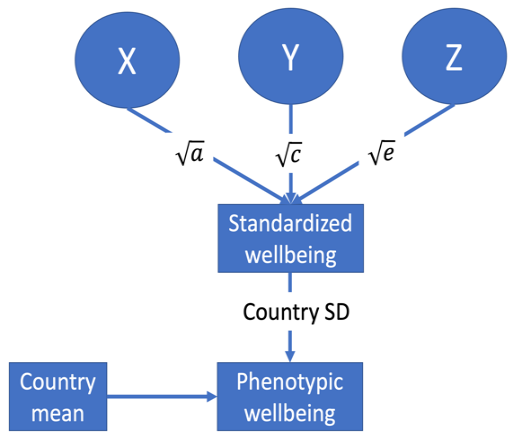
The figure illustrates the key components of the model, based on the classic twin model and incorporating means and standard deviations for each country.
- The code ensures that x1 (genes) and y1 (shared environments) are shared by both twins in an MZ-pair, while their unique environments are provided by z1 and z2.
- The code above is repeated (outer loop, see below) 100 times, in total generating 1000x157x100 values.
- Note that given the normal distribution variables as input, the main simulation approach yields some scores that fall outside the basic 0-10 range. However, >96% were within the range and all scores were allowed in the simulations. For robustness, analyses were rerun with the out-of-range scores truncated to the nearest value (0/10), and results were virtually identical.
- The full code for DZ twins and for other scenarios is provided at the end of the Supplementary material

**2) Additional scenario**

The main manuscript includes five alternative scenarios, to examine the robustness across different assumptions and situations: 1) Basic scenario with h^2^=.38 and n=1000 per country, 2) taking into account different population sizes across countries, 3) including regional differences in mean wellbeing, 4) using h^2^=.32 as input parameter, and 5) adjusting for random error.

Here, we provide the results from another scenario:

*Equal absolute genetic variance, but not equal relative genetic variance (heritability)*

All the main scenarios are based on the notion of the meta-analytic heritability estimates as representing a mean of within-country heritabilities globally. That is, the simulations allow for variation around the mean heritability, and the mean is based on the meta-analyses. An alternative approach would assume that the absolute genetic variance is similar across countries, so that if country A has an established heritability of .38 and country B has an unknown heritability (yet assumed same genetic variance as A) but twice the observed phenotypic variance (compared to A), then the additional variance in B would assumedly result from environmental sources and the heritability would be reduced to .38/2=.19 (i.e., 38/100=.38 and 38/200=.19). More specifically, the mean SD for the meta-analyses countries was 1.63, while the mean SD for countries worldwide was 2.23. In this scenario, the added variability in the countries not included in the meta-analyses would be assumed to result from larger E variance only. We ran simulations, based on scenario 1, but where the genetic variance was held similar across countries, by incorporating the mean of observed SDs of the countries contributing to the meta-analyses. This analysis yielded the following parameters:

A=.16 (.15-.17), C=.19 (.18-.19), E=.66 (.65-.66)

As the assumption of equal absolute genetic variance is rather extreme, we do not consider these specific results highly plausible. Yet, they are informative both with regards to the general pattern (including a substantial C-factor) and as estimating a boundary for the assumption of equal absolute vs relative genetic variance.

**3) Additional figure**

The main findings are presented as scatterplots, density plots and bar plots. As twin studies often present findings in a biometric diagram with latent factors A, C and E, here we also provide such a figure (based on scenario 3) so as to ease comparisons with other studies. Figure S1 shows the effects of the three major sources of worldwide wellbeing, with standardized effects of .65, .48, and .68 for the A, C and E factors respectively.


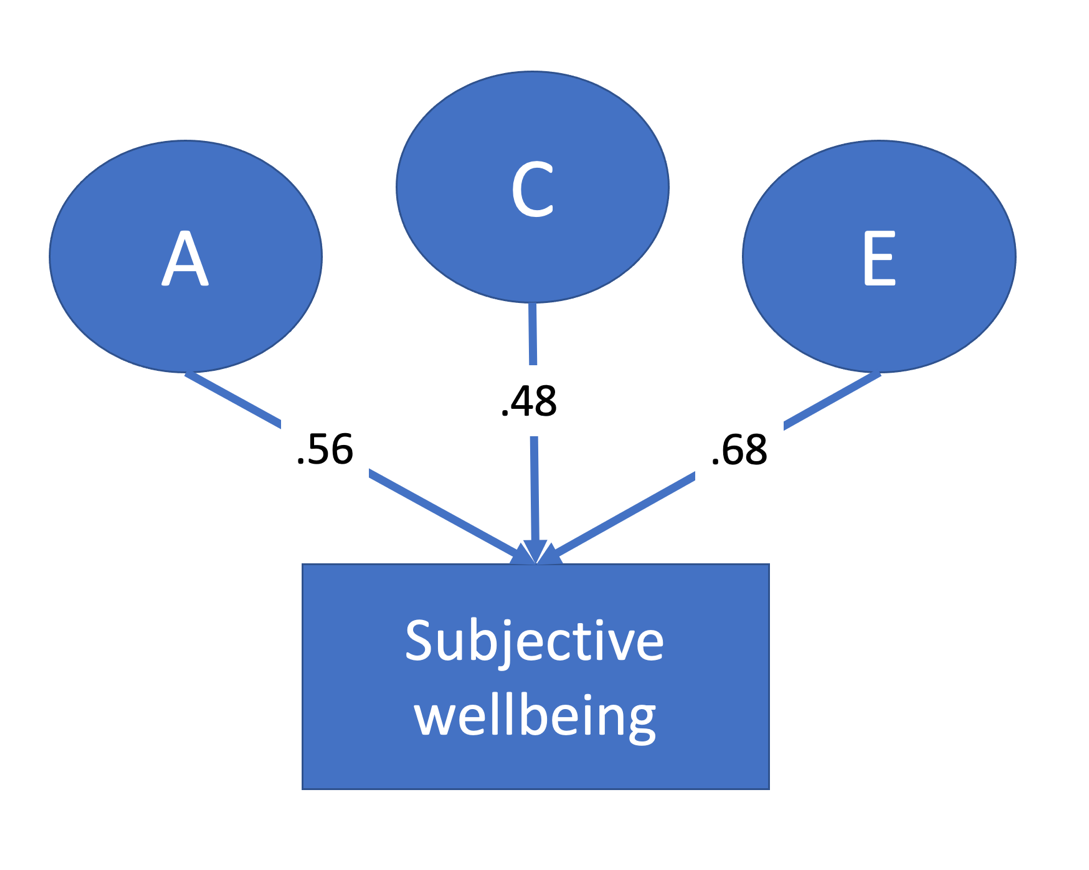


Figure S1. ACE model of worldwide wellbeing. A=Additive genetic factors; C=Shared (common) environment; and E=Non-shared (unique) environment. From scenario 3, five-year averages, 2015-2019.

**4) Country means and standard deviations**. Values from simulation, equal to observed values in the World Happiness Report. Scores are averages across the five-year period 2015-2019. (Note that the WHR typically report annual scores as mean of the three previous years).

| **#** | **Country** | **Mean** | **SD** |  | **#** | **Country** | **Mean** | **SD** |  | **#** | **Country** | **Mean** | **SD** |
| --- | --- | --- | --- | --- | --- | --- | --- | --- | --- | --- | --- | --- | --- |
| 1 | Afghanistan | 3.19 | 1.65 |  | 61 | Iran | 4.68 | 2.50 |  | 121 | Senegal | 4.83 | 2.06 |
| 2 | Albania | 4.75 | 2.66 |  | 62 | Iraq | 4.62 | 2.86 |  | 122 | Serbia | 5.67 | 2.29 |
| 3 | Algeria | 5.09 | 1.98 |  | 63 | Ireland | 7.03 | 1.78 |  | 123 | Sierra Leone | 4.30 | 3.06 |
| 4 | Argentina | 6.21 | 2.24 |  | 64 | Israel | 7.12 | 1.68 |  | 124 | Singapore | 6.36 | 1.49 |
| 5 | Armenia | 4.51 | 2.20 |  | 65 | Italy | 6.19 | 1.74 |  | 125 | Slovakia | 6.20 | 1.89 |
| 6 | Australia | 7.25 | 1.72 |  | 66 | Ivory Coast | 4.94 | 2.72 |  | 126 | Slovenia | 6.15 | 2.00 |
| 7 | Austria | 7.20 | 1.63 |  | 67 | Jamaica | 5.89 | 2.40 |  | 127 | Somalia | 5.01 | 2.20 |
| 8 | Azerbaijan | 5.19 | 1.64 |  | 68 | Japan | 5.89 | 1.89 |  | 128 | South Africa | 4.82 | 2.61 |
| 9 | Bahrain | 6.13 | 2.22 |  | 69 | Jordan | 4.92 | 2.68 |  | 129 | South Korea | 5.87 | 2.09 |
| 10 | Bangladesh | 4.62 | 2.21 |  | 70 | Kazakhstan | 5.93 | 1.94 |  | 130 | South Sudan | 3.26 | 2.95 |
| 11 | Belarus | 5.50 | 1.81 |  | 71 | Kenya | 4.50 | 2.59 |  | 131 | Spain | 6.38 | 1.70 |
| 12 | Belgium | 6.89 | 1.53 |  | 72 | Kosovo | 5.96 | 2.41 |  | 132 | Sri Lanka | 4.40 | 2.33 |
| 13 | Benin | 4.66 | 2.77 |  | 73 | Kuwait | 6.07 | 2.37 |  | 133 | Swaziland | 4.30 | 2.60 |
| 14 | Bhutan | 5.08 | 1.47 |  | 74 | Kyrgyzstan | 5.27 | 1.93 |  | 134 | Sweden | 7.34 | 1.65 |
| 15 | Bolivia | 5.77 | 2.12 |  | 75 | Laos | 4.89 | 2.17 |  | 135 | Switzerland | 7.54 | 1.63 |
| 16 | Bosnia & Herzegovina | 5.46 | 2.23 |  | 76 | Latvia | 5.93 | 1.71 |  | 136 | Syria | 3.46 | 2.81 |
| 17 | Botswana | 3.54 | 2.58 |  | 77 | Lebanon | 4.96 | 2.10 |  | 137 | Taiwan | 6.47 | 1.82 |
| 18 | Brazil | 6.38 | 2.49 |  | 78 | Lesotho | 3.71 | 3.00 |  | 138 | Tajikistan | 5.40 | 1.91 |
| 19 | Bulgaria | 5.00 | 2.01 |  | 79 | Liberia | 3.95 | 3.31 |  | 139 | Tanzania | 3.40 | 2.61 |
| 20 | Burkina Faso | 4.59 | 2.33 |  | 80 | Libya | 5.50 | 2.57 |  | 140 | Thailand | 6.05 | 2.13 |
| 21 | Burundi | 3.78 | 2.82 |  | 81 | Lithuania | 6.04 | 2.00 |  | 141 | Togo | 4.04 | 2.48 |
| 22 | Cambodia | 4.67 | 2.70 |  | 82 | Luxembourg | 7.08 | 1.39 |  | 142 | Trinidad & Tobago | 6.19 | 2.28 |
| 23 | Cameroon | 5.02 | 2.67 |  | 83 | Macedonia | 5.16 | 2.18 |  | 143 | Tunisia | 4.57 | 2.24 |
| 24 | Canada | 7.27 | 1.71 |  | 84 | Madagascar | 3.95 | 2.06 |  | 144 | Turkey | 5.30 | 2.22 |
| 25 | Central African Rep | 3.08 | 2.53 |  | 85 | Malawi | 3.59 | 2.87 |  | 145 | Turkmenistan | 5.40 | 1.40 |
| 26 | Chad | 4.33 | 2.78 |  | 86 | Malaysia | 5.70 | 1.93 |  | 146 | Uganda | 4.35 | 2.92 |
| 27 | Chile | 6.36 | 2.07 |  | 87 | Maldives | 5.20 | 2.09 |  | 147 | Ukraine | 4.33 | 2.11 |
| 28 | China | 5.20 | 2.17 |  | 88 | Mali | 4.55 | 2.37 |  | 148 | United Arab Em. | 6.75 | 2.16 |
| 29 | Colombia | 6.22 | 2.55 |  | 89 | Malta | 6.70 | 1.87 |  | 149 | United Kingdom | 6.97 | 1.66 |
| 30 | Comoros | 4.29 | 3.06 |  | 90 | Mauritania | 4.31 | 2.11 |  | 150 | United States | 6.90 | 1.94 |
| 31 | Congo (Brazzaville) | 4.88 | 3.05 |  | 91 | Mauritius | 5.98 | 2.32 |  | 151 | Uruguay | 6.42 | 2.22 |
| 32 | Congo (Kinshasa) | 4.25 | 2.02 |  | 92 | Mexico | 6.49 | 2.21 |  | 152 | Uzbekistan | 6.13 | 2.36 |
| 33 | Costa Rica | 7.07 | 2.16 |  | 93 | Moldova | 5.68 | 1.96 |  | 153 | Venezuela | 4.95 | 2.82 |
| 34 | Croatia | 5.43 | 1.74 |  | 94 | Mongolia | 5.28 | 1.92 |  | 154 | Vietnam | 5.22 | 1.78 |
| 35 | Cyprus | 5.94 | 2.29 |  | 95 | Montenegro | 5.42 | 2.29 |  | 155 | Yemen | 3.46 | 2.29 |
| 36 | Czech Republic | 6.79 | 1.71 |  | 96 | Morocco | 5.16 | 2.36 |  | 156 | Zambia | 4.09 | 2.91 |
| 37 | Denmark | 7.60 | 1.60 |  | 97 | Mozambique | 4.60 | 3.29 |  | 157 | Zimbabwe | 3.48 | 2.56 |
| 38 | Dominican Republic | 5.47 | 3.18 |  | 98 | Myanmar | 4.37 | 2.07 |  |  |  |  |  |
| 39 | Ecuador | 5.97 | 2.51 |  | 99 | Namibia | 4.57 | 2.58 |  |  |  |  |  |
| 40 | Egypt | 4.32 | 2.10 |  | 100 | Nepal | 5.00 | 2.74 |  |  |  |  |  |
| 41 | El Salvador | 6.24 | 2.61 |  | 101 | Netherlands | 7.44 | 1.32 |  |  |  |  |  |
| 42 | Estonia | 5.87 | 1.82 |  | 102 | New Zealand | 7.31 | 1.65 |  |  |  |  |  |
| 43 | Ethiopia | 4.31 | 1.95 |  | 103 | Nicaragua | 6.07 | 2.84 |  |  |  |  |  |
| 44 | Finland | 7.71 | 1.46 |  | 104 | Niger | 4.54 | 2.73 |  |  |  |  |  |
| 45 | France | 6.56 | 1.71 |  | 105 | Nigeria | 5.02 | 2.61 |  |  |  |  |  |
| 46 | Gabon | 4.79 | 2.40 |  | 106 | North Cyprus | 5.69 | 1.96 |  |  |  |  |  |
| 47 | Gambia | 4.73 | 3.03 |  | 107 | Norway | 7.53 | 1.60 |  |  |  |  |  |
| 48 | Georgia | 4.51 | 2.04 |  | 108 | Pakistan | 5.42 | 2.03 |  |  |  |  |  |
| 49 | Germany | 7.03 | 1.65 |  | 109 | Palestinian Terr. | 4.65 | 2.48 |  |  |  |  |  |
| 50 | Ghana | 4.79 | 2.50 |  | 110 | Panama | 6.33 | 2.66 |  |  |  |  |  |
| 51 | Greece | 5.49 | 2.21 |  | 111 | Paraguay | 5.68 | 2.27 |  |  |  |  |  |
| 52 | Guatemala | 6.41 | 2.86 |  | 112 | Peru | 5.73 | 2.51 |  |  |  |  |  |
| 53 | Guinea | 4.40 | 2.69 |  | 113 | Philippines | 5.74 | 2.60 |  |  |  |  |  |
| 54 | Haiti | 3.59 | 2.44 |  | 114 | Poland | 6.14 | 1.74 |  |  |  |  |  |
| 55 | Honduras | 5.67 | 3.06 |  | 115 | Portugal | 5.65 | 2.19 |  |  |  |  |  |
| 56 | Hong Kong | 5.51 | 1.80 |  | 116 | Qatar | 6.37 | 2.26 |  |  |  |  |  |
| 57 | Hungary | 5.76 | 1.86 |  | 117 | Romania | 6.02 | 2.17 |  |  |  |  |  |
| 58 | Iceland | 7.50 | 1.61 |  | 118 | Russia | 5.74 | 2.12 |  |  |  |  |  |
| 59 | India | 3.93 | 2.14 |  | 119 | Rwanda | 3.35 | 2.14 |  |  |  |  |  |
| 60 | Indonesia | 5.19 | 2.42 |  | 120 | Saudi Arabia | 6.41 | 2.37 |  |  |  |  |  |

**5) R-code for simulations**

# R-code for simulations in "Worldwide wellbeing: Simulated twins reveal genetic

# and (hidden) environmental influences” (Røysamb, Moffitt, Caspi, Ystrøm & Nes)

# Script developed by Espen Røysamb, 2021-2022

library(psych)

library(ggplot2)

library(readxl)

library(tidyr)

library(reshape2)

library(dplyr)

# Reading and prepearing data ----

WHR20tot <- read_excel("…/WHR20_DataForTable2.1.xls") # WHR data

Wpop <- read_excel(".../Worldwide population er3.xlsx", col_names = T) #Country populations

#Recode names

names(WHR20tot)[names(WHR20tot) == "year"] <- "Year"

names(WHR20tot)[names(WHR20tot) == "Country name"] <- "Country"

names(WHR20tot)[names(WHR20tot) == "Life Ladder"] <- "Ladder"

names(WHR20tot)[names(WHR20tot) == "Standard deviation of ladder by country-year"] <- "Sdladder"

#Select variables for further use

WHR20short <- WHR20tot[,c('Country','Year','Ladder','Sdladder')]

# Transforming from long to wide format, with mean (Ladder and Sdladder) across years for each Country

WHRwide <- dcast(WHR20short, Country ~ Year, value.var="Ladder")

WHRwideSD <- dcast(WHR20short, Country ~ Year, value.var="Sdladder")

# A) code for specific years - select one year manually

cantril <- subset(WHRwide, select=c('2019')) # selects specific year, means

cantrilsd <- subset(WHRwideSD, select=c('2019')) # selects specific year, sd

cantril <- subset(WHRwide, select=c('2018')) # selects specific year, means

cantrilsd <- subset(WHRwideSD, select=c('2018')) # selects specific year, sd

cantril <- subset(WHRwide, select=c('2017')) # selects specific year, means

cantrilsd <- subset(WHRwideSD, select=c('2017')) # selects specific year, sd

cantril <- subset(WHRwide, select=c('2016')) # selects specific year, means

cantrilsd <- subset(WHRwideSD, select=c('2016')) # selects specific year, sd

cantril <- subset(WHRwide, select=c('2015')) # selects specific year, means

cantrilsd <- subset(WHRwideSD, select=c('2015')) # selects specific year, sd

# B) code for mean of several years - not to be used if single years

cantril <- subset(WHRwide, select=c('2015','2016','2017', '2018', '2019'))

cantrilsd <- subset(WHRwideSD, select=c('2015','2016','2017', '2018', '2019'))

WHRwide$snitt <- rowMeans(cantril, na.rm = TRUE)

WHRwide$snittSD <- rowMeans(cantrilsd, na.rm = TRUE)

#Join WHR-file with population-file

WHRwidex <- left_join(WHRwide, Wpop, by="Country")

WHRwide3 <- na.omit(WHRwidex[, c('Country', 'snitt', 'snittSD', 'pop')]) # Valid countries retained

#Vectors for simulation

length_yr <- dim(WHRwide3)[1] ## NB number of cases for specific year

mwb <- WHRwide3$snitt #mean ladder score for n countries

sdwb <- WHRwide3$snittSD #sd for ladder score, per country

# Preparing for simulation - defining objects ----

## Includes alternative scenarios (with diff assumptions, for robustness).

scenario <- 1 # Choose according to scenario tested

a1 <- .38 # Heritability (within-country)

c1 <- 0.0 # Shared environment (within-country)

e1 <- (1-a1-c1) # Unique environment (within-country)

n <- 1000 # N of cases (twin pairs, each zygosity) per country

twin_sd <- 1.63 # For scenario 6 (Supp.) (mean sd in countries in twin meta-analysis)

nsim <- 100 # Number of simulations

#creating empty matrices

mz1 <- data.frame(matrix(NA, ncol = length_yr , nrow = n))

mz2 <- data.frame(matrix(NA, ncol = length_yr , nrow = n))

dz1 <- data.frame(matrix(NA, ncol = length_yr, nrow = n))

dz2 <- data.frame(matrix(NA, ncol = length_yr, nrow = n))

wb_mz_cor1_vec <- rep(NA,nsim)

wb_dz_cor1_vec <- rep(NA,nsim)

wb_mz_cor_vec <- rep(NA,nsim)

wb_dz_cor_vec <- rep(NA,nsim)

mz1longtot <- data.frame(matrix(NA, ncol = nsim, nrow = length_yr*n))

mz2longtot <- data.frame(matrix(NA, ncol = nsim, nrow = length_yr*n))

dz1longtot <- data.frame(matrix(NA, ncol = nsim, nrow = length_yr*n))

dz2longtot <- data.frame(matrix(NA, ncol = nsim, nrow = length_yr*n))

if (scenario == 2) {

n <- round(WHRwide3$pop / sum(WHRwide3$pop) *157000 , 0)

mz1longtot <- data.frame(matrix(NA, ncol = nsim, nrow = sum(n)))

mz2longtot <- data.frame(matrix(NA, ncol = nsim, nrow = sum(n)))

dz1longtot <- data.frame(matrix(NA, ncol = nsim, nrow = sum(n)))

dz2longtot <- data.frame(matrix(NA, ncol = nsim, nrow = sum(n)))

}

mz_corrs_within <- data.frame(matrix(NA, ncol = nsim , nrow = length_yr ))

dz_corrs_within <- data.frame(matrix(NA, ncol = nsim , nrow = length_yr ))

mz_cor <- rep(NA,length_yr)

dz_cor <- rep(NA,length_yr)

mzcorr_world_tot <- rep(NA, nsim)

dzcorr_world_tot <- rep(NA, nsim)

A_world <- rep(NA, nsim)

C_world <- rep(NA, nsim)

E_world <- rep(NA, nsim)

# Simulation - Loops ----

# j-loop repeats simulation nsim (e.g., 100) times

# i-loop repeats one set of simulations for each country

for(j in 1:nsim) {

# Scenario 1, with fixed mean heritability across countries

if (scenario == 1) {

## MZ twins

for(i in 1:length_yr) {

x <- rnorm(n,0,1)

y <- rnorm(n,0,1)

z1 <- rnorm(n,0,1)

z2 <- rnorm(n,0,1)

mz1[,i] <- as.vector(mwb[i] + sdwb[i]*scale(sqrt(a1)*x + sqrt(c1)*y + sqrt(e1)*z1))

names(mz1)[i] <- paste("m1_",i, sep="")

mz2[,i] <- as.vector(mwb[i] + sdwb[i]*scale(sqrt(a1)*x + sqrt(c1)*y + sqrt(e1)*z2))

names(mz2)[i] <- paste("m2_",i, sep="")

mz_cor[i] <- cor(mz1[i],mz2[i])

}

## DZ twins

for(i in 1:length_yr) {

x <- rnorm(n,0,1)

xu1 <- rnorm(n,0,1) #Unique genes for twin 1

xu2 <- rnorm(n,0,1) #Unique genes for twin 2

y <- rnorm(n,0,1)

z1 <- rnorm(n,0,1)

z2 <- rnorm(n,0,1)

dz1[,i] <- as.vector(mwb[i] + sdwb[i]*scale(sqrt(a1/2)*x + sqrt(a1/2)*xu1 + sqrt(c1)*y + sqrt(e1)*z1))

names(dz1)[i] <- paste("m1_",i, sep="")

dz2[,i] <- as.vector(mwb[i] + sdwb[i]*scale(sqrt(a1/2)*x + sqrt(a1/2)*xu2 +sqrt(c1)*y + sqrt(e1)*z2))

names(dz2)[i] <- paste("m2_",i, sep="")

dz_cor[i] <- cor(dz1[i],dz2[i])

}

} #end of scenario 1 sim

### Scenario 2, with population-based n's (i.e., proportion of world pop)

if (scenario == 2) {

## MZ twins

mz1 <- list(1:length_yr) # Use lists due to diff N's across countries

mz2 <- list(1:length_yr)

for(i in 1:length_yr) {

x <- rnorm(n[i],0,1)

y <- rnorm(n[i],0,1)

z1 <- rnorm(n[i],0,1)

z2 <- rnorm(n[i],0,1)

mz1[[i]] <- as.vector(mwb[i] + sdwb[i]*scale(sqrt(a1)*x + sqrt(c1)*y + sqrt(e1)*z1))

mz2[[i]] <- as.vector(mwb[i] + sdwb[i]*scale(sqrt(a1)*x + sqrt(c1)*y + sqrt(e1)*z2))

mz_cor[i] <- cor(as.numeric(unlist(mz1[i])) , as.numeric(unlist(mz2[i])))

}

## DZ twins

dz1 <- list(1:length_yr) # Use lists due to diff N's across countries

dz2 <- list(1:length_yr)

for(i in 1:length_yr) {

x <- rnorm(n[i],0,1)

xu1 <- rnorm(n[i],0,1) #Unique genes for twin 1

xu2 <- rnorm(n[i],0,1) #Unique genes for twin 2

y <- rnorm(n[i],0,1)

z1 <- rnorm(n[i],0,1)

z2 <- rnorm(n[i],0,1)

dz1[[i]] <- as.vector(mwb[i] + sdwb[i]*scale(sqrt(a1/2)*x + sqrt(a1/2)*xu1 + sqrt(c1)*y + sqrt(e1)*z1))

dz2[[i]] <- as.vector(mwb[i] + sdwb[i]*scale(sqrt(a1/2)*x + sqrt(a1/2)*xu2 +sqrt(c1)*y + sqrt(e1)*z2))

dz_cor[i] <- cor(as.numeric(unlist(dz1[i])) , as.numeric(unlist(dz2[i])))

}

} # End of scenario 2 sim

#Scenario 3 (regional diffs) is implemented by adjusting the c-factor above (i.e., c1 <- 0.05)

#Scenario 4 is implemented by changing the a-factor above (i.e., a1 <- 0.32)

#Scenario 5 (adj for measurement error) is calculated at end of script

### Scenario 6, with fixed mean genetic variance (differing h2 - Supplementary)

if (scenario == 6) {

## MZ twins

for(i in 1:length_yr) {

x <- rnorm(n,0,1)

y <- rnorm(n,0,1)

z1 <- rnorm(n,0,1)

z2 <- rnorm(n,0,1)

mz1[,i] <- as.vector(mwb[i] + sdwb[i]*scale(sqrt(a1*twin_sd^2)*x + sqrt(sdwb[i]^2-a1*twin_sd^2)*z1))

names(mz1)[i] <- paste("m1_",i, sep="")

mz2[,i] <- as.vector(mwb[i] + sdwb[i]*scale(sqrt(a1*twin_sd^2)*x + sqrt(sdwb[i]^2-a1*twin_sd^2)*z2))

names(mz2)[i] <- paste("m2_",i, sep="")

mz_cor[i] <- cor(mz1[i],mz2[i])

}

# DZ twins

for(i in 1:length_yr) {

x <- rnorm(n,0,1)

xu1 <- rnorm(n,0,1)

xu2 <- rnorm(n,0,1)

y <- rnorm(n,0,1)

z1 <- rnorm(n,0,1)

z2 <- rnorm(n,0,1)

dz1[,i] <- as.vector(mwb[i] + sdwb[i]*scale(sqrt(a1/2*twin_sd^2)*x + sqrt(a1/2*twin_sd^2)*xu1 + sqrt(sdwb[i]^2-a1*twin_sd^2)*z1))

names(dz1)[i] <- paste("m1_",i, sep="")

dz2[,i] <- as.vector(mwb[i] + sdwb[i]*scale(sqrt(a1/2*twin_sd^2)*x + sqrt(a1/2*twin_sd^2)*xu2 + sqrt(sdwb[i]^2-a1*twin_sd^2)*z2))

names(dz2)[i] <- paste("m2_",i, sep="")

dz_cor[i] <- cor(dz1[i],dz2[i])

}

} # End of scenario 3

### i-loops ended, now into j-loop

#stacking into long files

if (scenario == 2) {

mz1long <- matrix(unlist(mz1)) # Creates long-file (worldwide)

mz2long <- matrix(unlist(mz2))

dz1long <- matrix(unlist(dz1))

dz2long <- matrix(unlist(dz2))

mz1longtot[j] <- mz1long # Combines long files for all nsims

mz2longtot[j] <- mz2long

dz1longtot[j] <- dz1long

dz2longtot[j] <- dz2long

}

if (scenario == 1 | scenario == 6) {

mz1long <- stack(mz1)

mz2long <- stack(mz2)

dz1long <- stack(dz1)

dz2long <- stack(dz2)

mz1longtot[j] <- mz1long$values

mz2longtot[j] <- mz2long$values

dz1longtot[j] <- dz1long$values

dz2longtot[j] <- dz2long$values

}

mzcorr_world_tot[j] <- cor(mz1longtot[j], mz2longtot[j],use="complete" ) # mz-corr across countries

dzcorr_world_tot[j] <- cor(dz1longtot[j], dz2longtot[j],use="complete" ) # dz-corr across countries

A_world[j] <- 2*(mzcorr_world_tot[j] - dzcorr_world_tot[j]) # A for each of nsim sim

C_world[j] <- 2*dzcorr_world_tot[j] - mzcorr_world_tot[j] # C for each of nsim sim

E_world[j] <- 1 - A_world[j] - C_world[j]

mz_corrs_within[j] <- mz_cor

dz_corrs_within[j] <- dz_cor

}

### j-loop ends here

# Simulation results ----

## Results for individual countries ----

A_country_tot <- 2*(mz_corrs_within - dz_corrs_within) # Calculate A for each country for each sim

A_country_tot

colMeans(A_country_tot)

mean(colMeans(A_country_tot)) # Mean within-country heritability!

sd(colMeans(A_country_tot)) # SD of within-country heritability across nsim (i.e., SE)

sapply(A_country_tot, min) #minimum A across countries for each sim

mean(sapply(A_country_tot, min))

sapply(A_country_tot, max)

mean(sapply(A_country_tot, max))

C_country_tot <- 2* dz_corrs_within - mz_corrs_within # Calculate C for each country for each sim

C_country_tot

colMeans(C_country_tot)

mean(colMeans(C_country_tot)) # Mean within-country C!

sd(colMeans(C_country_tot)) # SD of within-country C across nsim (i.e., SE)

E_country_tot <- 1 - A_country_tot - C_country_tot # Calculate C for each country for each sim

E_country_tot

colMeans(E_country_tot)

mean(colMeans(E_country_tot)) # Mean within-country E!

sd(colMeans(E_country_tot)) # SD of within-country E across nsim (i.e., SE)

## Corrs worldwide ----

mean(mzcorr_world_tot)

mean(dzcorr_world_tot)

## Calculate means and CI for worldwide A, C & E ----

A_world_mean <- mean(A_world)

C_world_mean <- mean(C_world)

E_world_mean <- mean(E_world)

A_world_CI_l <- A_world_mean - 1.96*sd(A_world)

A_world_CI_u <- A_world_mean + 1.96*sd(A_world)

C_world_CI_l <- C_world_mean - 1.96*sd(C_world)

C_world_CI_u <- C_world_mean + 1.96*sd(C_world)

E_world_CI_l <- E_world_mean - 1.96*sd(E_world)

E_world_CI_u <- E_world_mean + 1.96*sd(E_world)

ACE_world <- matrix(c( A_world_mean, C_world_mean, E_world_mean,

A_world_CI_l, C_world_CI_l, E_world_CI_l,

A_world_CI_u, C_world_CI_u, E_world_CI_u),

nrow=3, dimnames = list(c("A", "C", "E"), c("Mean", "CI_lower", "CI_upper")))

## Final parameters with CI’s ----

round(ACE_world, 4 )

##Controlling for reliability ----

rel <- .80

A_world_rel <- A_world / rel

C_world_rel <- C_world / rel

E_world_rel <- (E_world -(1-rel)) / rel

world_rel <- data.frame(A_world_rel, C_world_rel, E_world_rel)

rowSums(world_rel)

A_world_mean_rel <- mean(A_world_rel)

C_world_mean_rel <- mean(C_world_rel)

E_world_mean_rel <- mean(E_world_rel)

A_world_CI_l_rel <- A_world_mean_rel - 1.96*sd(A_world_rel)

A_world_CI_u_rel <- A_world_mean_rel + 1.96*sd(A_world_rel)

C_world_CI_l_rel <- C_world_mean_rel - 1.96*sd(C_world_rel)

C_world_CI_u_rel <- C_world_mean_rel + 1.96*sd(C_world_rel)

E_world_CI_l_rel <- E_world_mean_rel - 1.96*sd(E_world_rel)

E_world_CI_u_rel <- E_world_mean_rel + 1.96*sd(E_world_rel)

ACE_world_rel <- matrix(c( A_world_mean_rel, C_world_mean_rel, E_world_mean_rel,

A_world_CI_l_rel, C_world_CI_l_rel, E_world_CI_l_rel,

A_world_CI_u_rel, C_world_CI_u_rel, E_world_CI_u_rel),

nrow=3, dimnames = list(c("A", "C", "E"), c("Mean", "CI_lower", "CI_upper")))

round(ACE_world_rel, 4 )
